# Suitable Combination of Direct Intensity Modulation and Spreading Sequence for LIDAR with Pulse Coding

**DOI:** 10.3390/s18124201

**Published:** 2018-11-30

**Authors:** Gunzung Kim, Yongwan Park

**Affiliations:** Department of Information and Communication Engineering, Yeungnam University, 280 Daehak-Ro, Gyeongsan, Gyeongbuk 38541, Korea; gzkim@yu.ac.kr

**Keywords:** LIDAR, time-of-flight, IM/DD OCDMA, free-space optical communication, modulation, spreading code

## Abstract

In the coded pulse scanning light detection and ranging (LIDAR) system, the number of laser pulses used at a given measurement point changes depending on the modulation and the method of spreading used in optical code-division multiple access (OCDMA). The number of laser pulses determines the pulse width, output power, and duration of the pulse transmission of a measurement point. These parameters determine the maximum measurement distance of the LIDAR and the number of measurement points that can be employed per second. In this paper, we suggest possible combinations of modulation and spreading technology that can be used for OCDMA, evaluate their performance and characteristics, and study optimal combinations according to varying operating environments.

## 1. Introduction

Pulse scanning light detection and ranging (LIDAR) measures the distance to a given object using a time-of-flight (ToF) technique that measures the time required for a pulse to transmit to and reflect off the object [1,2,3,4,5,6]. The distance image of the surroundings can be generated with excellent angular resolution, and is used to determine the area that can be traveled while mounted on an autonomous vehicle or an autonomous mobile robot. Many factors determine the operating characteristics of pulse scanning LIDAR, and can be divided into the characteristics of the transmission and generation of a pulse, and those of the reception of a reflected pulse [3,7,8]. In the transmitter, the pulse scanning LIDAR determines the wavelength of the laser as well as the pulse width, interval, and peak power [9]. In the receiver, it determines the size of the receiving aperture and uses a photodetector, a pulse detection method, the threshold-to-noise ratio (TNR), and a range estimation method [10,11,12,13]. The characteristics used to generate pulses in the transmitter are limited by the maximum permissible exposure (MPE) to comply with eye safety standards [14]. The most critical parameter that determines the maximum measurement distance in LIDAR is the pulse peak power of the transmitter and the TNR of the receiver. As the strength of the received signal is proportional to the peak power of the pulse and inversely proportional to the square of the measured distance, the higher the pulse peak power and the lower the TNR, the greater the distance that can be measured. Thus, if the characteristics of one parameter are improved, the characteristics of the other parameters worsen [9]. Depending on the primary purpose of LIDAR, one or two of the parameters are used as characteristics of preference, and the remaining are rendered MPE-compliant.

For pulsed, amplitude-modulated continuous wave (AMCW), and frequency-modulated continuous wave (FMCW) LIDAR, the maximum measurable range *R* depends on the ToF measurement techniques as follows:

(1) $$R=\left\{\begin{array}{cc} c\frac{T}{2} & \;\mathrm{for}\;a\;\mathrm{pulsed}\;\mathrm{LIDAR}\\ c\frac{\phi }{4\pi f} & \;\mathrm{for}\;\mathrm{an}\;\mathrm{amplitude}-\mathrm{modulated}\;\mathrm{continuous}\;\mathrm{wave}\;\mathrm{LIDAR}\\ c\frac{B}{2\lambda } & \;\mathrm{for}\;a\;\mathrm{frequency}-\mathrm{modulated}\;\mathrm{continuous}\;\mathrm{wave}\;\mathrm{LIDAR} \end{array}\right.$$

where *c* is the speed of light, *T* is the pulse repetition period of the pulsed LIDAR, ϕ is the phase shift between the transmitted and reflected signal, *f* is the modulation frequency of the AMCW LIDAR, *B* is the frequency sweep bandwidth of FMCW LIDAR, and λ is the frequency shift per unit of time [2,4,5]. *T* limits the maximum range of the pulsed LIDAR, ϕ and *f* limit the maximum range of the AMCW LIDAR, and *B* and λ limit the maximum range of FMCW LIDAR. The main drawback of pulsed scanning LIDAR is that its maximum measurable range is proportional to the maximum pulse repetition period, and high-angular-resolution scanning is only possible at low revolutions per second. In pulsed scanning LIDAR, we can measure the target distance at greater than 100 m with a pulse repetition period higher than 0.666 μs using Equation (1). For these scanning LIDAR, we can calculate the minimum range resolution δR by the following equation [9,15].

(2) $$\delta R=\left\{\begin{array}{cc} c\frac{1}{2W} & \;\mathrm{for}\;a\;\mathrm{pulsed}\;\mathrm{LIDAR}\\ c\frac{1}{2\phi } & \;\mathrm{for}\;\mathrm{an}\;\mathrm{amplitude}-\mathrm{modulated}\;\mathrm{continuous}\;\mathrm{wave}\;\mathrm{LIDAR}\\ c\frac{1}{2B} & \;\mathrm{for}\;a\;\mathrm{frequency}-\mathrm{modulated}\;\mathrm{continuous}\;\mathrm{wave}\;\mathrm{LIDAR} \end{array}\right.$$

where *W* is the pulse width of the pulsed LIDAR, ϕ is the phase shift of the AMCW LIDAR, and *B* is the frequency sweep bandwidth of the FMCW LIDAR. The range resolution is the ability to distinguish the reflections from two targets that are very close in range. The time difference δt between the reflections from two targets is proportional to the range resolution. When the pulse repetition period is smaller than the time difference between the reflections, the range ambiguity problem occurs. In order to solve this problem only, increasing the pulse repetition period decreases the range ambiguity by increasing the maximum distance, but increases the idle listening time between the laser pulse transmission and reception and then reduces the number of measurement points, frame refresh rate, and angular resolution per second [9,16,17].

In the pulsed scanning LIDAR system, range ambiguity resolution extends the system’s unambiguous range between transmit pulses at a high pulse repetition frequency (PRF). Some studies have focused on solving the range ambiguity of pulse scanning LIDAR by using pulse coding to avoid crosstalk [16,17,18,19,20] or mutual interference that occurs when two or more LIDARs simultaneously operate [16,17,21]. Such LIDARs measure distances using multiple pulses generated by random sequences [18,19,20] or specially designed codes [16,17,21], rather than one pulse per measurement point. By adapting the intensity-modulated direct detection (IM/DD) optical code-division multiple access (OCDMA, a kind of specially designed code) method to the emitted laser pulses of a scanning LIDAR system [16,17], these pulses become orthogonal to other pulses and then they are distinguished from each other [22,23,24,25,26,27,28]. In the receiver, all of the reflected pulses that are received at the same time are individually identified and demodulated into original information by the receiver. Thus, the reflected waves of laser pulses emitted from different measurement points can be received at the same time. As a result, we can ignore the idle listening time and eliminate the range ambiguity problem, and then we can achieve longer maximum distance, high measurement point, high frame refresh rate, and high angular resolution per second at the same time.

**Figure 1:**
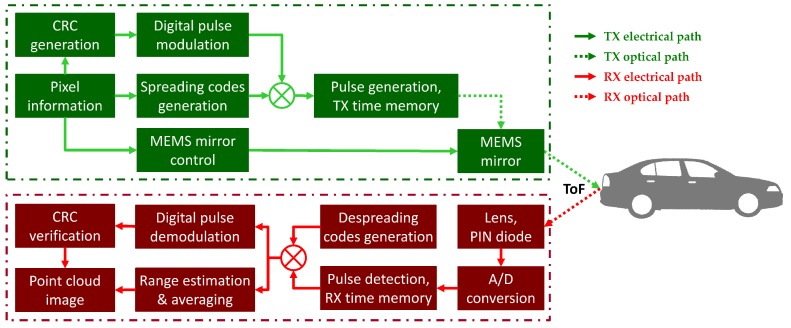
Overall architecture and operation flow of the proposed scanning light detection and ranging (LIDAR) system. A/D: Analog-to-digital; CRC: Cyclic redundancy check; MEMS: Microelectromechanical systems; PIN: Positive–intrinsic–negative; RX: Receiver; ToF: Time-of-flight; TX: Transmitter.

The proposed scanning LIDAR consists of a transmitter and a receiver, which operate independently of each other, as shown in Figure 1. The transmitter codes the pixel location bit stream through the IM/DD OCDMA technique and generates laser pulses using a laser diode. Subsequently, the transmitter emits the laser pulses generated in a bearing direction using a scanning-based microelectromechanical systems (MEMS) scanning mirror. The receiver digitalizes the received reflected pulses using an analog-to-digital converter (ADC) and demodulates them using the IM/DD OCDMA technique. The receiver then calculates the ToFs of the laser pulses and converts them to distances to the relevant objects based on the emission time of the pixel locations. Even if multiple pulses are used, the parameters that determine the characteristics of the transmitter in the conventional pulse scanning scheme are maintained. To comply with eye safety standards, the pulse peak power is distributed across several pulses so that the energy allocated to a pulse decreases in inverse proportion to the number of pulses, and the time required to transmit pulses at a given measurement point is proportional to the number of pulses [16,17,21]. The use of multiple pulses also enhances the accuracy of the distance measurement [17]. In LIDAR with pulse coding [16,17], the number of pulses used at a measurement point is determined by the modulation method and the spreading code method.

The optical channel differs significantly from radio frequency (RF) channels. Unlike RF systems, where the amplitude, frequency, and phase of the carrier signal are modulated, the intensity of the optical carrier is modulated in optical systems. In an optical wireless communication system using unipolar signaling, the numbers and positions of the pulses to be transmitted and empty slots are determined by the modulation and spreading code methods used [24,27,29,30,31]. To accurately demodulate and despread the signal at the receiver of the code pulse LIDAR, both the numbers and positions of the pulses and the empty slots are used. In a unipolar optical communication system, on–off keying (OOK), pulse position modulation (PPM), differential PPM (DPPM), multipulse PPM (MPPM), digital pulse interval modulation (DPIM), and dual-header pulse interval modulation (DH-PIM) are widely used as modulation techniques, and prime code (PC) and optical orthogonal code (OOC) are widely used as spreading code techniques [29,30,31].

In this paper, we investigate the characteristics of various modulations and spreading code methods that can be used for the prototype LIDAR with pulse coding and compare various characteristics of LIDAR according to the combinations. The prototype LIDAR system uses a unipolar optical digital modulation scheme and spreading code to identify pixel locations and determine the distance to an object [16,17]. The number of pulses, pulse peak power, average signal-to-noise ratio (SNR), maximum measurable distance, accuracy and precision of the measured distance, and system error probability vary depending on the combination of modulation scheme and spreading code scheme used. At each pixel, the prototype LIDAR system generates pixel information to identify the measuring point and emission time. Pixel information is represented by a nine-bit stream consisting of a leading or trailing ‘1’, a five-bit column identification number (CID), and a three-bit cyclic redundancy check (CRC) checksum. The CID represents the locations of corresponding pixels for each measurement angle and identifies each of the 30 columns from a 30 × 30 range image. The pixel information is converted to a sequence of pulses by the selected optical modulation scheme and spreading code technique. The transmitter adjusts the angle of the MEMS mirror based on the pixel information, emits and deflects the optically modulated and spread laser pulses in the desired bearing direction, and simultaneously records its row identification number (RID), CID, and emission time. The receiver collects and digitizes the reflected pulses, and then despreads and demodulates them to the pixel information. The prototype system has its operation timing adjusted so that CIDs are not overwrapped on reception. Through the receiving process, the CID included in the received reflected pulses can be used to identify the RID and emission time [16]. A prototype LIDAR system comprises commercial off-the-shelf (COTS) products [32], such as an optical modulator module, an amplified photodetector module, an MEMS mirror development kit, an ADC evaluation module, a digital signal processor (DSP) with an ARM processor evaluation kit, and a Windows PC. We used an OPM-LD-D1-C digital high-speed pulsed laser generator as the optical modulator [33], which is designed for systems that require high-speed transmission and operates at up to 1 GHz, with a peak current of 500 mA and a peak optical power of 250 mW. The coded laser pulses were deflected and steered in the desired measurement angle using a two-axis MEMS mirror from Mirrorcle Technologies, Inc. [34,35] that has a aluminum-coated mirror with a diameter of 1.2 mm. An ET-4000AF from EOT [36,37] that operates at frequencies of up to 9 GHz was chosen for the high-speed amplified positive–intrinsic–negative (PIN) gallium arsenide (GaAs) photodiode equipped with a transimpedance amplifier (TIA) that senses light levels as low as 100 nW. We selected an ADC12J4000 from Texas Instruments (TI), which is a 12 bit, 4 GHz radio frequency-sampling ADC with a buffered analog input [38,39].

The outline of the paper is organized as follows. In Section 2, the popular unipolar optical digital modulation schemes and spreading codes are reviewed. A performance evaluation of combinations of unipolar optical digital modulation schemes and spreading codes is explained in Section 3. Finally, Section 4 draws the conclusions reached.

## 2. Unipolar Optical Digital Modulation Schemes and Spreading Codes

### 2.1. Unipolar Optical Digital Modulation Schemes

**Figure 2:**
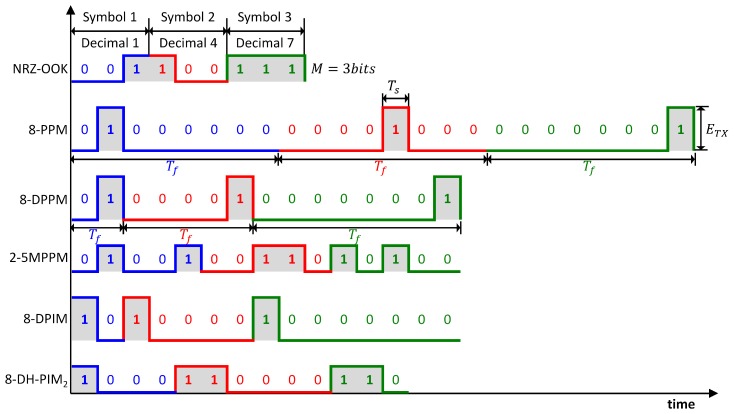
Time waveforms for on–off keying (OOK), pulse position modulation (PPM), differential PPM (DPPM), multipulse PPM (MPPM), digital pulse interval modulation (DPIM), and dual-header pulse interval modulation (DH-PIM) signals. The different symbols for the different modulation schemes shown in the figure are denoted by different colors.

Because the average optical power of LIDAR is constrained, it is useful to determine a modulation scheme that can provide the requisite bandwidth and use power efficiently. The unipolar optical digital modulation scheme converts a symbol to a digital data stream that is composed of pulses and empty slots. The symbol is represented as a bit sequence called a block and the size of the block is the number of bits. Many digital modulation schemes have been proposed for use in optical wireless communication systems. Given the requirements, the performance of a communication system depends on how the information is represented in the modulation scheme. The types of modulation are thus the critical determinants of the system design. In digital modulation schemes, information is embedded in both mark and space slots, which are generated in terms of a fixed time slot. Each discrete amplitude of a modulated signal appears by varying the characteristics of the pulse at a discrete time. Time characteristics such as pulse position, width, and spacing are modulated using the instantaneous modulation signal, but a constant sampling frequency is sustained. The OOK provides higher bandwidth efficiency, but poor optical power performance. Digital pulse time modulation (DPTM) techniques such as PPM, DPPM, MPPM, DPIM, and DH-PIM are recognized as block codes through the OOK, which provides a balance between bandwidth and optical power efficiency [31]. Digital modulation schemes can be divided into two main categories: Isochronous and anisochronous. In an isochronous mode, the length of the symbol is fixed. In the anisochronous mode, the length of the symbol is variable. The OOK, PPM, and MPPM are isochronous, whereas the DPPM, DPIM, and DH-PIM are anisochronous. An illustration of the overall conversion method and the time waveforms of modulation techniques with fixed pulse width ($T_{s}$) and fixed time slot rate ($R_{s}$) that are discussed—the OOK, PPM, DPPM, MPPM, DPIM, and DH-PIM—are shown in Figure 2. *M* is the size of the input block, $E_{TX}$ is the pulse peak power, $T_{f}=T_{s}L_{max}=\frac{L_{max}}{R_{s}}$ is the block duration, $T_{s}$ is the time slot duration, $R_{s}$ is the time slot rate, and $L_{max}$ is the maximum number of time slots.

OOK is the predominant pulse modulation format in optical wireless communication systems. It uses the simple method of amplitude-shift keying (ASK) modulation that represents digital data depending on the presence of an optical pulse [31,40,41,42]. In its simplest form, the presence of a pulse for a particular bit duration is represented by ‘1’, and its absence for the same bit duration is represented by ‘0’. OOK can either be return to zero (RZ) or non-return to zero (NRZ). In NRZ–OOK, the pulses fill the entire bit duration; and in RZ–OOK, they occupy a particular portion of the bit duration. Owing to the relatively wide pulse, NRZ–OOK has higher bandwidth efficiency, but lower power efficiency than RZ–OOK. In the OOK, symbols are displayed as amplitude pulse groups. A combination of an *M*-bit input block with symbols for on or off can represent $L=2^{M}$ unique combinations. Three significant advantages of the OOK are that it provides a high SNR, low distortion performance, and superior system linearity.

In PPM, each bit of an *M*-bit input block is mapped to one of $L=2^{M}$ possible symbols [31,40,41,42,43,44]. A frame consists of a pulse that occupies a slot, and the remaining slots have no pulse. Therefore, the information is displayed as a pulse position within the same symbol as the decimal value of the *M*-bit input block. Because PPM requires both slot and symbol synchronization at the receiver to demodulate the signal, it delivers impressive optical power performance, but at the cost of bandwidth and circuit simplicity.

In DPPM, an *M*-bit input block maps to one of $L=2^{M}$ unique DPPM symbols, including L−1 empty slots and a pulse [40,41,42,44,45,46]. The DPPM symbol is derived from the corresponding PPM symbol by removing all empty slots following the pulse, thus reducing the average symbol length and increasing bandwidth efficiency. DPPM indicates its own symbol synchronization when all symbols end with a pulse. For a long sequence of zeros, there may be a slot synchronization problem that can be handled using a guard slot (GS) immediately after the pulse is removed. The DPPM improves bandwidth and power efficiency over the PPM for a fixed average bit rate and fixed available bandwidth.

As the level of coding increases, the number of PPM slots and the required transmission bandwidth both increase exponentially. To overcome these limitations, MPPM was introduced as a way to improve the bandwidth utilization of PPM. MPPM is a generalization of PPM that allows more than one pulse per symbol. Moreover, *w*-pulse *n*-slot MPPM has nw unique symbols that correspond to filling *n* slots with *w* pulses in a frame [40,42,43,44,47,48,49,50,51,52]. This approach reduces the bandwidth to half of that in the traditional PPM at the same transmission efficiency. That is, a single frame can carry information of size log2nw bits. By contrast, for PPM, this rate is $\log_{2}L$ bits. The amount of information that the MPPM can transfer increases with the number of pulses in the fixed-length frame. The disadvantage is that if one or more of these pulses are erroneous, the frame is incorrectly demodulated. Therefore, too many source bits are affected. MPPM provides half the information capacity of the PPM, and is inferior to it in terms of error performance.

DPIM has built-in symbol synchronization that improves bandwidth efficiency and data speed compared to PPM and power efficiency compared to OOK [31,40,41,42,44]. The waveform of DPIM is similar to that of DPPM, except the variable frame length and the pulse are located at the beginning of the frame. In DPIM, each symbol starts with a pulse of short duration after the optional GS, followed by the number of empty time slots, which is determined by the decimal value of the bit input block. In other words, a symbol is represented by a discrete interval between consecutive pulses belonging to two consecutive frames. The GS consists of zero or more empty slots, and is vital to avoiding continuous pulses when the input symbol is zero. The frame length of the DPIM may vary depending on the bit input block. The number of DPIM and DPPM slots increases exponentially with OOK bit rates as bit resolution increases. If two systems are included in the GS, this increase is even greater. As the slot frequency increases, bandwidth requirements also increase.

In DH-PIM, a symbol consists of two sections: A heading that starts a symbol and an ending information section. The *n*th symbol $S_{n}\left(h_{n},d_{n}\right)$ starts with the header $h_{n}$ of duration $T_{h}=\left(\alpha +1\right)T_{s}$ and ends with the sequence of $d_{n}$ empty slots, where α>0 is an integer [31,40,41]. Depending on the most significant bit (MSB) of the input block, two headers are considered, $H_{1}$ and $H_{2}$, corresponding to MSB=0 and MSB=1, respectively. $H_{1}$ and $H_{2}$ have pulses of $0.5\alpha T_{s}$ and $\alpha T_{s}$, respectively. Each pulse is followed by a GS of appropriate length $T_{g}\in \left\{\left(\frac{\alpha }{2}+1\right)T_{s},T_{s}\right\}$. The value of empty slots $d_{n}\in \left\{0,1,\cdots ,2^{M-1}-1\right\}$ is the decimal value of the input block if the symbol starts with $H_{1}$. If the symbol starts with $H_{2}$, it is the decimal value of the 1’s complement of the input code word. The header pulses play the dual role of symbol initiation and time reference for the preceding and succeeding symbols, resulting in built-in symbol synchronization. In other words, DH-PIM creates a symbol to enable built-in symbol synchronization. Thus, like the DPPM symbol, the DH-PIM removes the extra time slot after the pulse and increases the average symbol length compared with PIM, thus increasing data throughput.

Comparisons of modulation techniques with fixed pulse width are based on various parameters, such as bandwidth occupancy, distortion, SNR, suitability for transmission channels, and error probability. No scheme yields optimal performance and negotiates all signals. For optical transmission, DPTMs are preferred because of their high pulse peak power and low average power characteristics. They require higher bandwidth than OOK, and provide a higher SNR. If *M*-bits are needed to present a symbol, OOK requires *M* time slots and maximum *M* pulses, but DPTM requires $2^{M}$ time slots and one or two pulses. In the case of LIDAR, since the laser pulse reflected from the object is received, as shown in Equation (3), the higher the pulse peak power used for transmission, the longer the distance that can be measured. The pulse peak power is limited by the MPE, so the smaller the number of pulses, the higher the pulse peak power that can be used. Therefore, LIDAR can measure a longer distance by adopting a method such as DPTM, in which the number of pulses necessary for symbol representation is small. The disadvantage of DPTM is that it requires symbol synchronization and, therefore, more circuitry and complexity than other approaches, which are outside of the scope of this paper. Table 1 and Table 2 [31,40,41,42,43,44,45,46,47,50,51] summarize the characteristics of *M*-bit input blocks when they are converted into symbols by OOK, PPM, DPPM, MPPM, DPIM, and DH-PIM, where $R_{s}$ is the time slot rate and $N_{0}$ is the energy of noise. In the case of optical communication, the influence of path loss can be ignored. The maximum transmitted energy is calculated using the received average energy. The received energy per bit, received energy per symbol, and power efficiency are calculated using the maximum transmitted energy. By contrast, in the case of LIDAR, the maximum energy to be emitted is fixed, and the reflected signal from the object is received. Thus, the influence of path loss must be reflected in the received energy. Therefore, in this paper, energy $E_{TX}$ emitted from LIDAR is reflected off the surface of an object at a distance *R* away, and the received energy $E_{RX}$ is calculated by Equation (3). $\tau_{o}$ is the optics transmission; $\tau_{a}$ is the atmospheric transmission; $D_{R}$ is the receiver aperture diameter; $\rho_{T}$ is the target surface reflectivity; and $\theta_{R}$ is the target surface angular dispersion. The received energy per bit $E_{b}$ and received energy per symbol $E_{s}$ are calculated by Table 2. Table 3 [31,40,43,44,45,47,50,51,53,54,55] summarizes the error probability of the digital pulse modulation techniques. The symbol error rate (SER) $P_{se}$ is calculated by Equation (4) and Table 3, and the packet error rate (PER) $P_{pe}$ by Equation (5) and Table 3 using $E_{RX}$, which is the received energy according to each modulation technique. $P_{0}$ is the probability of ‘0’, $P_{1}$ is the probability of ‘1’, $P_{\epsilon 0}$ is the marginal probability of ‘0’, $P_{\epsilon 1}$ is the marginal probability of ‘1’, Q–function Q(x) is the probability that a standard Gaussian random variable takes a value larger than *x*, *M* is the size of the bit block, $N_{pkt}$ is the number of bits in a packet, and $\bar{L}$ is the average symbol length. The packet is a sequence of the pulses and the empty slots that is dedicated to a measurement point and generated by the modulation scheme. The SER $P_{se}$ is optimum when the threshold factor *k* is 0.5.

(3) $$E_{RX}=E_{TX}\frac{\pi \tau_{o}\tau_{a}^{2}D_{R}^{2}\rho_{T}}{4R^{2}\theta_{R}}$$

(4) $$P_{se}=P_{0}P_{\epsilon 0}+P_{1}P_{\epsilon 1}=P_{0}Q\left(k\sqrt{\frac{E_{s}}{2N_{0}}}\right)+\left(1-P_{0}\right)Q\left(\left(1-k\right)\sqrt{\frac{E_{s}}{2N_{0}}}\right)$$

(5) $$P_{pe}=1-{\left(1-P_{se}\right)}^{\frac{N_{pkt}\bar{L}}{M}}\approx \frac{N_{pkt}\bar{L}}{M}P_{se}$$

### 2.2. One-Dimensional Optical Spreading Codes

Time-division multiple access (TDMA), wavelength-division multiple access (WDMA), and OCDMA are techniques of multiple access in optical wireless communications that implement multiplexed transmission and multiple access. Of these, OCDMA supports simultaneous multiple transmissions at the same frequency and the same time slot, and it uses optical spreading codes so that multiple users can be separately identified without interfering with one another. In the simplest type of optical spreading code, one-bit period $T_{B}$ is divided into *M* time chips with duration $T_{C}=\frac{T_{B}}{M}$, and these *M* chips are filled with optically spread code. That is, the spreading code sequence is selected to characterize the maximum auto-correlation and minimum cross-correlation to optimize the difference between a correct signal and interference. Primary time spreading codes suitable for OCDMA schemes are OOCs and various PC families. These are very sparse codes, and their code weights are small, thus requiring a long transmission time after spreading. In optical spreading codes, the weight is the number of ones in each of its codewords and the most important parameters in characterizing spreading codes, such as the number of chips, pulse peak power, and error probability [27].

OOCs are generally expressed as a quadruple $\left(N,w,\lambda_{a},\lambda_{c}\right)$, where *N* is the code length, *w* is code weight (i.e., the number of ones), $\lambda_{a}$ is the upper bound of the autocorrelation value for a non-zero shift, and $\lambda_{c}$ is the upper limit of the cross-correlation value [29,56,57,58]. In the OOC, a particular case where $\lambda_{a}=\lambda_{c}=\lambda$ is expressed by the optimal OOC (N,w,λ). |C| represents the cardinality of the OOC family (i.e., the size of the code set as the number of codewords in the code set). For OOCs to satisfy the condition $\lambda_{a}=\lambda_{c}=\lambda =1$, |C| is upper-bounded by $\left[C\right]<\left\lfloor \frac{N-1}{w(w-1)}\right\rfloor$, where the ⌊x⌋ equation denotes the largest integer less than or equal to *x*. Various algorithms can generate OOC codes that satisfy this condition. By default, the unipolar sequences generated by these algorithms can all be assumed to be OOC code sets, as long as the code set correlation constraints are met. The code generation of OOC (N,3,1) and OOC (31,3,1) are shown in Table 4 and Table 5, respectively.

Compared to that of OOC, the PC generation process is relatively simple. A code set with a code length of $n=p^{2}$ and code weight w=p has *p* unique sequences [24,27,29]. An example of a PC set with p=5 is shown in Table 6. The main disadvantage of PC is that the number of available codes is limited. The code length of PC is only $p^{2}$, which may affect the system’s performance in terms of bit error rate (BER) and multiple access interference (MAI) [24,27]. Therefore, longer codes that maintain desirable properties are beneficial.

As the cardinality of the PC corresponds to the number of concurrent users, *M*, it is equal to the *w* of the PC, and *w* is equal to the prime number *p*. Thus, *p* must be increased. To increase the number of users on the network, the weight *w* must be greater. A modified prime code (MPC) has been proposed to overcome the drawbacks of the PC [24,27,29,59]. This optical sequence eliminates some redundant pulses from the original PC with a pulse, assuming a BER requirement such as $10^{-9}$ and a certain number of users. The weight of the MPC is smaller than that of the PC, but the code can support the *p* group containing *p* sequences and $p^{2}$ subscribers having the same code sequence length as $p^{2}$. The configuration of MPC is as follows: Generate the PC with *p* codewords. Any p−w pulse is removed from this PC, and the remaining pulses form a new code with a constant weight *w*. The length, weight, and cardinality of the MPC are *n*, w<p, and |C|=p, respectively. An example of an MPC set with p=5 and w=4 is shown in Table 7.

Table 8 summarizes the characteristics of OOC, PC, and MPC, including length, weight, peak auto-correlation, peak cross-correlation, cardinality, and bit error probability ($P_{sc}$), where *M* is the number of concurrent users [24,27,29,56,57,58,59,60,61,62,63,64,65,66].

## 3. Performance Evaluation of Combinations of Modulation and Spreading Code Techniques

### 3.1. Combinations of Modulation and Spreading Code Techniques

Table 9 shows the eight symbols that can be expressed in three-bit blocks according to the OOK, PPM, DPPM, MPPM, DPIM, and DH-PIM. The OOK, PPM, and MPPM have a fixed number of slots regardless of symbol values, and the DPPM, DPIM, and DH-PIM vary in the number of slots according to symbol values. The OOK, PPM, and MPPM can know the transmitted symbol by detecting the start and end of the pulse. As all three know the end if they detect the start, they should add a leading ‘1’ to indicate the start of the transmission before the first symbol. As the DPPM ends with ‘1’, the symbol can be known by the number of ‘0’s transmitted before ‘1’ is reached. The DPIM and DH-PIM can identify symbols with a number of ‘0’s after a ‘1’, and cannot know the symbols because the number of ‘0’s in the last symbol is unknown. In this case, we should mark the end of the transmission by appending a trailing ‘1’ to the end of the last symbol. We used zero GS for the modulation techniques because optical spreading codes are very sparse codes, and two or more successive ‘0’s precede a very sparse ‘1’.

The possible modulation schemes according to the size of the bit input block are shown in Table 10. As slot size increases, the number of slots required for modulation increase linearly in the OOK, but those in the PPM, DPPM, DPIM, and DH-PIM increase exponentially, and that of the MPPM increases exponentially but relatively mildly.

If the bit input block is partitioned into several block sizes according to Table 10, the number of slots according to each modulation scheme is as shown in Table 11. In the block partitioning column, the OOK, PPM, DPPM, and MPPM use a leading ‘1’ to indicate the start of transmission, and the DPIM and DH-PIM use a trailing ‘1’ to indicate its end. Splitting the bit input block into several smaller partitions requires fewer slots to be transferred than using a single large partition. However, as the number of ‘1’s for transmitting pulses is determined according to the number of partitions, the number of pulses to be transmitted increases when a plurality of small partitions is used and decreases when a large partition is used. All these block partitionings are used to evaluate the performance (e.g., the maximum distance, accuracy, and precision). In these block partitionings, **1**:4:4, **1**:5:3, **1**:6:2, and **1**:7:1 have equal numbers of ‘1’s and different numbers of ‘0’s, and then they have very similar performance. Therefore, block partitioning **1**:4:4 and **1**:5:3 were selected as two representatives of four three-small-piece block partitionings. The prototype LIDAR system is a non-directional non-line of sight (NLOS) optical wireless communication system that uses Lambertian diffusion. Eye safety is a critical issue in optical wireless systems because optical signals can penetrate the human cornea and potentially cause thermal damage to the retina. Optical transmitters must comply with the class 1 of the International Electrotechnical Commission (IEC) standard. The MPE is the highest power or energy density of a light source considered safe (i.e., less likely to cause damage).

The combination of modulation and spreading code techniques was determined to satisfy all operating conditions of the prototype LIDAR. The following operating characteristics were determined according to the combinations:

- Symbol stream;
- Block size and partitioning;
- Pulse peak power;
- Number of time slots;
- Number of pulses;
- Leading ‘1’ or trailing ‘1’.

The operating environment of the prototype system was simplified for the validation and performance evaluation. The length of the optical spreading code was proportional to the square of the cardinality so that the number of pulses used at one measurement point also increased in proportion to the square. To measure dozens or hundreds of measurement points at the same time, the cardinality of the spreading code must be very large. Therefore, to reduce the number of pulses used at one measurement point, the cardinality was lowered, and the number of simultaneous measurement points was reduced to shorten the spreading code length. If the bit input block were divided into partitions of various sizes and the optical spreading code with a cardinality of five were applied, the transmission characteristics would be as shown in Table 12 and Table 13. The number of time slots needed for transmission is the greatest, and each pair relates the number of slots, the number of pulses, and the maximum pulse output. The transmission power of the pulse is inversely proportional to the number of transmitted pulses.

The number of time slots $L_{max}$, the number of pulses $N_{p}$, and the pulse peak power $E_{TX}$ were determined for each measurement point depending on the combinations of block partitioning, modulation scheme, and spreading code. According to combinations of these techniques, the $L_{max}$ and $N_{p}$ used for each measurement point are shown in Table 12, and $E_{TX}$ is described in Table 13. OOK allocates one time slot per bit, so the number of time slots was constant regardless of the partitioning of the block. PPM, DPPM, and DPIM were different from each other in their numbers of ‘1’s and their average block sizes, but their number of time slots was the same according to the maximum block size, indicating whether a transmission is possible within a given time. These three modulation methods are identical regarding the parameters required to measure the performance of the LIDAR system, even though the symbol representation is different. DH-PIM had the advantage that the average and maximum block sizes were both smaller than those of PPM, DPPM, and DPIM. However, it had the disadvantage that the number of ‘1’s required for representing a block was large. If the number of slots corresponding to ‘1’ was larger, the pulse peak power was smaller. Unlike other modulation methods, in which the number of ‘1’s is fixed, DH-PIM changes the number of ‘1’s according to the block. MPPM had the advantage that the average and maximum block sizes were the smallest among the modulation schemes. However, as in the case of DH-PIM, the number of ‘1’s needed to represent a symbol was increased, so the pulse peak power was reduced.

The combination of using OOK as the modulation technique and PC or MPC as the spreading code technique had the smallest number of time slots. In this case, the number of time slots was always 225, regardless of the size of the block partition. The highest number of time slots was required when the block was divided into eight bits, the PPM was used as the modulation method, and the OOC was used as the spreading code technique. In this case, a total of 7969 time slots were required, and since one time slot was allocated 5 ns, 39.835 μs were required to complete the transmission. In this worst case, the transmission was completed within the allowed 67 μs of the prototype LIDAR system, so a combination of all possible modulation and spreading code techniques is possible. The combination of PPM, DPPM, or DPIM as the modulation technique and using OOC as the spreading code technique had the smallest number of ‘1’s. They required a small emission time and the largest pulse peak power. In Section 3.2, Table 11, Table 12 and Table 13 are used to evaluate the performance (i.e., maximum distance, accuracy, and precision) of possible combinations of optical modulation schemes and spreading code techniques that can be used for the prototype LIDAR system.

### 3.2. Performance Evaluation of Combined Techniques

**Figure 3:**
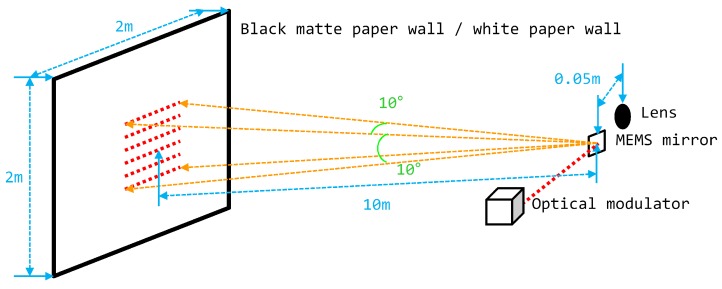
Experimental conditions and optical structure of the prototype LIDAR systems.

The experimental environment was the same as that for the prototype LIDAR system, and a modulation technique and a spreading code technique were used. Experiments were conducted using various parameters in Section 3.1 with a 2 m × 2 m white paper wall, as shown in Figure 3. We evaluated the performance of the following elements based on combinations of various modulation and spreading code techniques, as well as the operating characteristics of the prototype LIDAR system. As listed below, several operating conditions were specified according to the characteristics of the prototype LIDAR system [16,17,32] and simplified in order to compare only the performance of combinations:

- A nine-bit block was used to identify each measurement point, and the first bit was always ‘1’;
- Combination of modulation and spreading code: Table 11, Table 12 and Table 13;
- Up to five measurement points could be measured simultaneously;
- Pulse width was fixed at 5 ns and pulse transmission was completed within 67 μs;
- The maximum output of the laser pulse was eye-safety class 1 compliant;
- The maximum desired distance: 150 m;
- Range gate: 1 μs;
- Probability of false alarm: 0.5;
- False alarm rate: 500,000/s;
- TNR: 9.8 dB.

The prototype LIDAR system uses optical communication technology in LIDAR, and therefore evaluates its performance concerning both the characteristics of LIDAR and those of wireless communication. As LIDAR is a distance-measuring device, the maximum distance obtained is shown in Table 14, and the accuracy and precision are shown in Table 15 and Table 16, respectively. The maximum distance was determined by the TNR [3,5,10,11,17,67]. Using the result of the measured power ($E_{RX}$), the relationship between the received power and measured distance, and target surface reflectivity ($\rho_{T}$) illustrated in Equation (3), we estimated the received power based on the distance (*R*). Since detection is possible when the received power according to the distance is larger than the TNR, the longest distance having a received power that was larger than the TNR was regarded as the maximum distance [17]. Accuracy and precision were determined using the American Society for Photogrammetry and Remote Sensing (ASPRS)’s positional standards for digital elevation data [68,69]. We calculated the ground truth distance from the geographical relationship between the wall and the prototype LIDAR system. The distance error was calculated by comparing the measured results with the prototype LIDAR and the ground truth. In a non-vegetated terrain, the corresponding estimates of accuracy at the 95% confidence level were computed using ASPRS positional accuracy standards such that it was approximated by multiplying the root-mean-square-error (RMSE) by 1.96 to estimate the positional accuracy. The precision was equal to the standard deviation of the measurements. The maximum measurement distance of the pulse was proportional to the number of transmitted pulses, as were accuracy and precision.

Among the LIDAR performance indices, maximum distance, accuracy, and precision were most affected by the number of pulses used at one measurement point. Therefore, the maximum distance, accuracy, and precision were very similar when the same number of pulses was used at a measurement point due to the combination of modulation scheme and spreading code. Therefore, PPM, DPPM, and DPIM showed similar results, and MPPM and DH-PIM showed similar results. If the number of slots corresponding to ‘1’ was large, the pulse peak power was small, and the maximum measurement distance was shortened, but the accuracy and precision were improved. DH-PIM changes the number of ‘1’s according to the block. If the pulse peak power can be changed dynamically according to the number of ‘1’s, a longer pulse distance can be measured using the pulse peak power when the number of ‘1’s is small. However, as in the case of DH-PIM, the number of ‘1’s needed to represent a symbol was increased, so the pulse peak power was reduced. As a result, the maximum measurement distance was shortened, but accuracy and precision were improved. MPPM has the advantage that the average and maximum block sizes were the smallest among the modulation schemes. Compared to other modulation methods, MPPM exhibited the best balance of measurement distance, precision, and accuracy.

**Figure 4:**
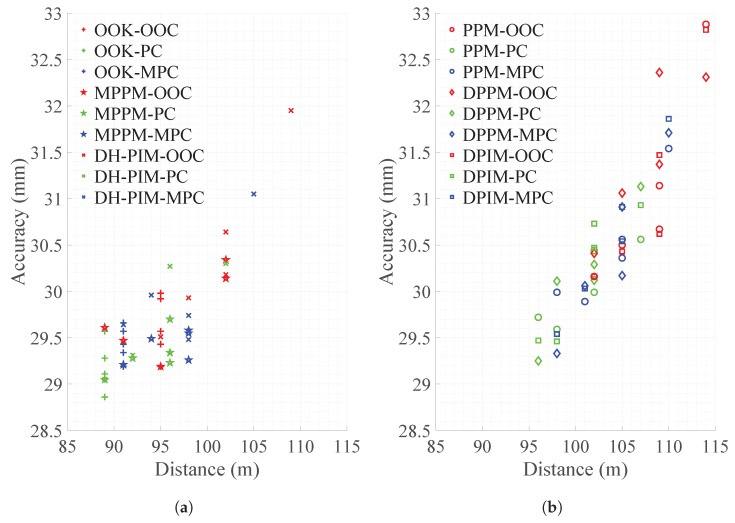
Relationship between the maximum distance and accuracy: (**a**) the combination of using OOK, MPPM, and DH-PIM; (**b**) the combination of using PPM, DPPM, and DPIM.

When the number of pulses was the smallest, the maximum measurement distance difference was 25 m, the accuracy difference was 3.2 mm, and the precision difference was 4.87 mm. In evaluating the performance of the LIDAR system, the maximum measurement distance was given priority over accuracy and precision, and the difference between the accuracy and the precision according to the modulation technique was negligible. Therefore, in the LIDAR system, it is best to use the combination with the lowest number of pulses satisfying the maximum allowable transmission time. Of the combinations we evaluated, the combination of using PPM, DPPM, or DPIM as the modulation technique and using OOC as the spreading code technique could measure the farthest distance. The relationship between maximum distance and accuracy by the combination of modulation schemes and spreading codes can be divided into two groups with similar trends. One group is indicated as OOK, MPPM, and DH-PIM, shown in Figure 4a, and Figure 4b shows the other group as PPM, DPPM, and DPIM.

As the prototype LIDAR system applies the communication scheme, the system error probability by Equation (6) according to the combination of the modulation scheme and the spreading technique used for transmission is shown in Table 17. The PER $P_{pe}$ of the modulation method by Equation (5) and Table 3 was calculated using the received energy $E_{RX}$ by Equation (3), reflected from the object at the maximum distance $R_{max}$ in Table 14. The bit error probability according to the spreading code $P_{sc}$ was calculated using Table 8. Additional parameters required for the calculation were used from Table 12, Table 13 and Table 14. If the received data had an error, the measured distance for the relevant measurement point was ignored. The combination of all the modulation and spreading codes we evaluated showed a very low error rate. As long as the number of points to be measured does not exceed the cardinality of the spreading code, the current combinations can be used reliably.

(6) $$P_{sys}=P_{sc}-\left(1-P_{sc}\right)P_{pe}$$

## 4. Conclusions

In the case of LIDAR with pulse coding, the pulse peak power and the maximum measurable distance both increase inversely proportionally to the number of transmitted pulses in order to comply with eye safety standards, and accuracy and precision increase in proportion to the number of pulses. Therefore, dividing the bit input block into several smaller partitions reduces transmission time and the maximum measurement distance, and improves accuracy and precision. Conversely, dividing the bit input block into large partitions increases the transfer time and maximum measurement distance, but reduces precision and accuracy. It is, thus, useful to select a modulation and a spread coding scheme according to the use and conditions of operation of LIDAR. If we need to measure distances even if accuracy and precision are low, we should use a combination of the smallest number of pulses and the smallest number of slots to increase the number of measurement points per second. If accuracy and precision are prioritized, the combination with the largest number of pulses is preferable.

## Figures and Tables

**Table 1:** Comparison of basic characteristics of digital pulse modulation techniques.

Modulation	Number of Bits per Block(*M*)	Maximum Number of Pulses ($N_{p}$)	Number of Possible Unique Symbols (*L*)	Maximum Number of Time Slots ($L_{\max}$)	Average Symbol Length ($\bar{L}$)	Slot Duration ($T_{s}$)	Bandwidth Requirements ($B_{\mathrm{req}}$)
NRZ-OOK	*M*	*M*	$2^{M}$	*M*	*M*	$\frac{1}{R_{s}}$	$R_{s}$
PPM	*M*	1	$2^{M}$	$L_{PPM}$	$L_{PPM}$	$\frac{1}{R_{s}}$	$\frac{MR_{s}}{L_{PPM}}$
DPPM	*M*	1	$2^{M}$	$L_{DPPM}$	$\frac{L_{DPPM}+1}{2}$	$\frac{1}{R_{s}}$	$\frac{2MR_{s}}{L_{DPPM}+1}$
MPPM	$\left\lfloor \log_{2}L\right\rfloor$	*w*	nw	*n*	*n*	$\frac{1}{R_{s}}$	$\frac{MR_{s}}{n}$
DPIM	*M*	1	$2^{M}$	$L_{DPIM}$	$\frac{L_{DPIM}+1}{2}$	$\frac{1}{R_{s}}$	$\frac{2MR_{s}}{L_{DPIM}+1}$
DH-PIM	*M*	2	$2^{M}$	$2^{M-1}+\alpha$	$\frac{2^{M-1}+2\alpha +1}{2}$	$\frac{1}{R_{s}}$	$\frac{2MR_{s}}{2^{M-1}+2\alpha +1}$

**Table 2:** Comparison of power characteristics of digital pulse modulation techniques.

Modulation	Peak-To-Average Power Ratio of Symbol (PAPR)	Peak Current of a Symbol ($I_{p}$)	Energy of a Symbol ($E_{s}$)	Energy of a Bit ($E_{b}$)
NRZ-OOK	2	$2{\bar{E}}_{RX\_OOK}$	$\frac{4{\bar{E}}_{RX\_OOK}^{2}}{R_{s}}$	$\frac{4{\bar{E}}_{RX\_OOK}^{2}}{R_{s}}$
PPM	$L_{PPM}$	$L_{PPM}{\bar{E}}_{RX\_PPM}$	$\frac{L_{PPM}^{2}{\bar{E}}_{RX\_PPM}^{2}}{R_{s}}$	$\frac{L_{PPM}^{3}{\bar{E}}_{RX\_PPM}^{2}}{MR_{s}}$
DPPM	$\frac{L_{DPPM}+1}{2}$	$\frac{\left(L_{DPPM}+1\right){\bar{E}}_{RX\_DPPM}}{2}$	$\frac{{\left(L_{DPPM}+1\right)}^{2}{\bar{E}}_{RX\_DPPM}^{2}}{4R_{s}}$	$\frac{{\left(L_{DPPM}+1\right)}^{3}{\bar{E}}_{RX\_DPPM}^{3}}{8MR_{s}}$
MPPM	$\frac{n}{w}$	$\frac{n{\bar{E}}_{RX\_MPPM}}{w}$	$\frac{n^{2}{\bar{E}}_{RX\_MPPM}^{2}}{w^{2}R_{s}}$	$\frac{n^{3}{\bar{E}}_{RX\_MPPM}^{3}}{w^{2}MR_{s}}$
DPIM	$\frac{L_{DPIM}+1}{2}$	$\frac{\left(L_{DPIM}+1\right)barE_{RX\_DPIM}}{2}$	$\frac{{\left(L_{DPIM}+1\right)}^{2}{\bar{E}}_{RX\_DPIM}^{2}}{4R_{s}}$	$\frac{{\left(L_{DPIM}+1\right)}^{3}{\bar{E}}_{RX\_DPIM}^{3}}{8MR_{s}}$
DH-PIM	$\frac{2\left(2^{M-1}+2\alpha +1\right)}{3\alpha }$	$\frac{2\left(2^{M-1}+2\alpha +1\right){\bar{E}}_{RX\_DH-PIM}}{3\alpha }$	$\frac{4{\left(2^{M-1}+2\alpha +1\right)}^{2}{\bar{E}}_{RX\_DH-PIM}^{2}}{9\alpha^{2}R_{s}}$	$\frac{2{\left(2^{M-1}+2\alpha +1\right)}^{3}{\bar{E}}_{RX\_DH-PIM}^{3}}{9\alpha^{2}MR_{s}}$

**Table 3:** Comparison of error probabilities of digital pulse modulation techniques.

Modulation	Probability of ‘0’ ($P_{0}$)	Probability of ‘1’ ($P_{1}$)	Marginal Probability of ‘0’($P_{\epsilon 0}$)	Optimum Symbol Error Probability ($P_{\mathrm{se}-\mathrm{opt}}$)
NRZ-OOK	$\frac{1}{2}$	$\frac{1}{2}$	$Q\left(\frac{k{\bar{E}}_{RX\_OOK}}{\sqrt{N_{0}R_{s}}}\right)$	$Q\left(\frac{{\bar{E}}_{RX\_OOK}}{2\sqrt{N_{0}R_{s}}}\right)$
PPM	$\frac{L_{PPM}-1}{L_{PPM}}$	$\frac{1}{L_{PPM}}$	$Q\left(\frac{kL_{PPM}{\bar{E}}_{RX\_PPM}}{\sqrt{N_{0}R_{s}}}\right)$	$Q\left(\frac{L_{PPM}{\bar{E}}_{RX\_PPM}}{2\sqrt{N_{0}R_{s}}}\right)$
DPPM	$\frac{L_{DPPM}-1}{L_{DPPM}+1}$	$\frac{2}{L_{DPPM}+1}$	$Q\left(\frac{k\left(L_{LPPM}+1\right){\bar{E}}_{RX\_DPPM}}{2\sqrt{N_{0}R_{s}}}\right)$	$Q\left(\frac{\left(L_{DPPM}+1\right){\bar{E}}_{RX\_DPPM}}{4\sqrt{N_{0}R_{s}}}\right)$
MPPM	$\frac{n-w}{n}$	$\frac{w}{n}$	$Q\left(\frac{kn{\bar{E}}_{RX\_MPPM}}{w\sqrt{N_{0}R_{s}}}\right)$	$Q\left(\frac{n{\bar{E}}_{RX\_MPPM}}{4w\sqrt{N_{0}R_{s}}}\right)$
DPIM	$\frac{L_{DPIM}-1}{L_{DPIM}+1}$	$\frac{2}{L_{DPIM}+1}$	$Q\left(\frac{k\left(L_{DPIM}+1\right){\bar{E}}_{RX\_DPIM}}{2\sqrt{N_{0}R_{s}}}\right)$	$Q\left(\frac{\left(L_{DPIM}+1\right){\bar{E}}_{RX\_DPIM}}{4\sqrt{N_{0}R_{s}}}\right)$
DH-PIM	$\frac{4{\bar{L}}_{DH-PIM}-3\alpha }{{\bar{L}}_{DH-PIM}}$	$\frac{3\alpha }{{\bar{L}}_{DH-PIM}}$	$Q\left(\frac{2k\left(2^{M-1}+2\alpha +1\right){\bar{E}}_{RX\_DH-PIM}}{3\alpha \sqrt{N_{0}R_{s}}}\right)$	$Q\left(\frac{\left(2^{M-1}+2\alpha +1\right){\bar{E}}_{RX\_DH-PIM}}{3\alpha \sqrt{N_{0}R_{s}}}\right)$

**Table 4:** Optical orthogonal code (OOC) (N,3,1) sequence indices for various lengths.

*N*	Sequence Index, When N≤49
7	{1,2,4}
13	{1,2,5},{1,3,8}
19	{1,2,6},{1,3,9},{1,4,11}
25	{1,2,7},{1,3,10},{1,4,12},{1,5,14}
31	{1,2,8},{1,3,12},{1,4,16},{1,5,15},{1,6,14}
37	{1,2,12},{1,3,10},{1,4,18},{1,5,13},{1,6,19},{1,7,13}
43	{1,2,20},{1,3,23},{1,4,16},{15,14},{1,6,17},{1,7,15},{1,8,19}

**Table 5:** OOC (31,3,1) sequences.

Index	Sequence Code
{1,2,8}	1100000100000000000000000000000
{1,3,12}	1010000000010000000000000000000
{1,4,16}	1001000000000001000000000000000
{1,5,15}	1000100000000010000000000000000
{1,6,14}	1000010000000100000000000000000

**Table 6:** Prime code (PC) sequences when p=5.

Groups	*i*					PC Sequence	PC Sequence Code
*x*	0	1	2	3	4				
0	0	0	0	0	0	$S_{0}$	$C_{0}=10000\;10000\;10000\;10000\;10000$
1	0	1	2	3	4	$S_{1}$	$C_{1}=10000\;01000\;00100\;00010\;00001$
2	0	2	4	1	3	$S_{2}$	$C_{2}=10000\;00100\;00001\;01000\;00010$
3	0	3	1	4	2	$S_{3}$	$C_{3}=10000\;00010\;01000\;00001\;00100$
4	0	4	3	2	1	$S_{4}$	$C_{4}=10000\;00001\;00010\;10000\;01000$

**Table 7:** Modified prime code (MPC) sequences $S_{i}^{\prime }$ constructed for p=5 and w=4.

Groups	*i*				MPC Sequence	MPC Sequence Code
*x*	$a_{0}$	$a_{1}$	$a_{2}$	$a_{3}$				
0	0	0	0	0	$S_{0}^{\prime }$	$C_{0}^{\prime }=10000\;10000\;10000\;10000\;00000$
1	0	1	2	3	$S_{1}^{\prime }$	$C_{1}^{\prime }=10000\;01000\;00100\;00010\;00000$
2	0	2	4	1	$S_{2}^{\prime }$	$C_{2}^{\prime }=10000\;00100\;00001\;01000\;00000$
3	0	3	1	4	$S_{3}^{\prime }$	$C_{3}^{\prime }=10000\;00010\;01000\;00001\;00000$
4	0	4	3	2	$S_{4}^{\prime }$	$C_{4}^{\prime }=10000\;00001\;00010\;10000\;00000$

**Table 8:** Performance comparison of optical spreading codes.

Characteristics	OOC (N,w,1)	PC	MPC
Length	*N*	$p^{2}$	$p^{2}$
Weight	*w*	*p*	*w*
Peak auto-correlation	1	*p*	*w*
Peak cross-correlation	1	1	1
Cardinality	$\left\lfloor \frac{N-1}{w(w-1)}\right\rfloor$	*p*	*p*
Bit error probability ($P_{sc}$)	12∑i=0w(−1)iwi1−iw2NM−1	12∑i=0p(−1)ipi1−i2pM−1	12∑i=0w2(−1)iw2i1−iw22p2M−1

**Table 9:** Three-bit block representation according to modulation technique.

Source Symbol	OOK	8-PPM	8-DPPM	2-5MPPM	8-DPIM	8-DH-PIM${}_{2}$
0	000	10000000	1	10001	1	100
1	001	01000000	01	01100	10	1000
2	010	00100000	001	01001	100	10000
3	011	00010000	0001	10010	1000	100000
4	100	00001000	00001	11000	10000	110000
5	101	00000100	000001	00101	100000	11000
6	110	00000010	0000001	00011	1000000	1100
7	111	00000001	00000001	10100	10000000	110

**Table 10:** Possible modulation schemes according to the size of the bit input block. Each cell expresses the modulated results as a 2-tuple $\left(N_{p},L_{max}\right)$, where $N_{p}$ is the maximum number of pulses and $L_{max}$ is the maximum number of time slots.

Block Size (*M*)	*M*-OOK	*M*-PPM	*M*-DPPM	2-*n*MPPM	*M*-DPIM	*M*-DH-PIM${}_{2}$
1-bit	1, 1	1, 2	1, 2	2, 3	1, 2	2, 3
2-bit	2, 2	1, 4	1, 4	2, 4	1, 4	2, 4
3-bit	3, 3	1, 8	1, 8	2, 5	1, 8	2, 6
4-bit	4, 4	1, 16	1, 16	2, 6	1, 16	2, 10
5-bit	5, 5	1, 32	1, 32	2, 9	1, 32	2, 18
6-bit	6, 6	1, 64	1, 64	2, 12	1, 64	2, 34
7-bit	7, 7	1, 128	1, 128	2, 17	1, 128	2, 66
8-bit	8, 8	1, 256	1, 256	2, 24	1, 256	2, 129
9-bit	9, 9	1, 512	1, 512	2, 33	1, 512	2, 258

**Table 11:** Possible block partitioning according to block partitioning and modulation techniques. A bold ‘**1**’ shows a leading ‘1’ or a trailing ‘1’. Each cell expresses the modulated results as a 2-tuple $\left(N_{p},L_{max}\right)$.

Block Paritioning	OOK	PPM	DPPM	MPPM	DPIM	DH-PIM${}_{2}$
**1**:2:2:2:2	9, 9	5, 17	5, 17	9, 17		
2:2:2:2:**1**					5, 17	9, 17
**1**:2:3:3	9, 9	4, 21	4, 21	7, 15		
2:3:3:**1**					4, 21	7, 17
**1**:4:4	9, 9	3, 33	3, 33	5, 13		
4:4:**1**					3, 33	5, 21
**1**:5:3	9, 9	3, 41	3, 41	5, 15		
5:3:**1**					3, 41	5, 25
**1**:6:2	9, 9	3, 69	3, 69	5, 17		
6:2:**1**					3, 69	5, 29
**1**:7:1	9, 9	3, 131	3, 131	5, 21		
7:1:**1**					3, 131	5, 70
**1**:8	9, 9	2, 257	2, 257	3, 25		
8:**1**					2, 257	3, 131

**Table 12:** Number of pulses $N_{p}$ and time slots $L_{max}$ as a combination of block partitioning, modulation, and spreading code. Each cell expresses the modulated and spread results as a 2-tuple $\left(N_{p},L_{max}\right)$.

Spreading Codes	OOK	PPM	DPPM	MPPM	DPIM	DH-PIM${}_{2}$
Block partitioning	**1**:2:2:2:2	**1**:2:2:2:2	**1**:2:2:2:2	**1**:2:2:2:2	2:2:2:2:**1**	2:2:2:2:**1**
OOC (31,3,1)	27,279	15,527	15,527	27,527	15,527	27,527
PC p=5	45,225	25,425	25,425	45,425	25,425	45,425
MPC p=5,w=4	36,225	20,425	20,425	36,425	20,425	36,425
Block partitioning	**1**:2:3:3	**1**:2:3:3	**1**:2:3:3	**1**:2:3:3	2:3:3:**1**	2:3:3:**1**
OOC (31,3,1)	27,279	12,651	12,651	21,465	12,651	21,527
PC p=5	45,225	20,525	20,525	35,375	20,525	35,425
MPC p=5,w=4	36,225	16,525	16,525	28,375	16,525	28,425
Block partitioning	**1**:4:4	**1**:4:4	**1**:4:4	**1**:4:4	4:4:**1**	4:4:**1**
OOC (31,3,1)	27,279	9,1023	9,1023	15,403	9,1023	15,651
PC p=5	45,225	15,825	15,825	25,325	15,825	25,525
MPC p=5,w=4	36,225	12,825	12,825	20,325	12,825	20,525
Block partitioning	**1**:5:3	**1**:5:3	**1**:5:3	**1**:5:3	5:3:**1**	5:3:**1**
OOC (31,3,1)	27,279	9,1271	9,1271	15,465	9,1271	15,775
PC p=5	45,225	15,1025	15,1025	25,375	15,1025	25,625
MPC p=5,w=4	36,225	12,1025	12,1025	20,375	12,1025	20,625
Block partitioning	**1**:8	**1**:8	**1**:8	**1**:8	8:**1**	8:**1**
OOC (31,3,1)	27,279	6,7967	6,7967	15,775	6,7967	9,4061
PC p=5	45,225	10,6425	10,6425	25,625	10,6425	15,3275
MPC p=5,w=4	36,225	8,6425	8,6425	20,625	8,6425	12,3275

**Table 13:** Pulse peak power $E_{TX}$ as a combination of block partitioning, modulation, and spreading code.

Spreading Codes	OOK	PPM	DPPM	MPPM	DPIM	DH-PIM${}_{2}$
Block partitioning	**1**:2:2:2:2	**1**:2:2:2:2	**1**:2:2:2:2	**1**:2:2:2:2	2:2:2:2:**1**	2:2:2:2:**1**
OOC (31,3,1)	8.9143 nJ	10.3254 nJ	10.3254 nJ	8.9143 nJ	10.3254 nJ	8.9143 nJ
PC p=5	7.8456 nJ	9.0895 nJ	9.0895 nJ	7.8456 nJ	9.0895 nJ	7.8456 nJ
MPC p=5,w=4	8.2957 nJ	9.6088 nJ	9.6088 nJ	8.2957 nJ	9.6088 nJ	8.2957 nJ
Block partitioning	**1**:2:3:3	**1**:2:3:3	**1**:2:3:3	**1**:2:3:3	2:3:3:**1**	2:3:3:**1**
OOC (31,3,1)	8.9143 nJ	10.9178 nJ	10.9178 nJ	9.4923 nJ	10.9178 nJ	9.4923 nJ
PC p=5	7.8456 nJ	9.6088 nJ	9.6088 nJ	8.4543 nJ	9.6088 nJ	8.4543 nJ
MPC p=5,w=4	8.2957 nJ	10.1601 nJ	10.1601 nJ	8.8336 nJ	10.1601 nJ	8.8336 nJ
Block partitioning	**1**:4:4	**1**:4:4	**1**:4:4	**1**:4:4	4:4:**1**	4:4:**1**
OOC (31,3,1)	8.9143 nJ	11.7319 nJ	11.7319 nJ	10.3254 nJ	11.7319 nJ	10.3254 nJ
PC p=5	7.8456 nJ	10.3254 nJ	10.3254 nJ	9.0895 nJ	10.3254 nJ	9.0895 nJ
MPC p=5,w=4	8.2957 nJ	10.9178 nJ	10.9178 nJ	9.6088 nJ	10.9178 nJ	9.6088 nJ
Block partitioning	**1**:5:3	**1**:5:3	**1**:5:3	**1**:5:3	5:3:**1**	5:3:**1**
OOC (31,3,1)	8.9143 nJ	11.7319 nJ	11.7319 nJ	10.3254 nJ	11.7319 nJ	10.3254 nJ
PC p=5	7.8456 nJ	10.3254 nJ	10.3254 nJ	9.0895 nJ	10.3254 nJ	9.0895 nJ
MPC p=5,w=4	8.2957 nJ	10.9178 nJ	10.9178 nJ	9.6088 nJ	10.9178 nJ	9.6088 nJ
Block partitioning	**1**:8	**1**:8	**1**:8	**1**:8	8:**1**	8:**1**
OOC (31,3,1)	8.9143 nJ	12.9835 nJ	12.9835 nJ	10.3254 nJ	12.9835 nJ	11.7319 nJ
PC p=5	7.8456 nJ	11.4269 nJ	11.4269 nJ	9.0895 nJ	11.4269 nJ	10.3254 nJ
MPC p=5,w=4	8.2957 nJ	12.0825 nJ	12.0825 nJ	9.6088 nJ	12.0825 nJ	10.9178 nJ

**Table 14:** Maximum distance $R_{max}$ as a combination of modulation and spreading code.

Spreading Codes	OOK	PPM	DPPM	MPPM	DPIM	DH-PIM ${}_{2}$
Block partitioning	**1**:2:2:2:2	**1**:2:2:2:2	**1**:2:2:2:2	**1**:2:2:2:2	2:2:2:2:**1**	2:2:2:2:**1**
OOC (31,3,1)	95 m	102 m	102 m	95 m	102 m	95 m
PC p=5	89 m	96 m	96 m	89 m	96 m	89 m
MPC p=5,w=4	91 m	98 m	98 m	91 m	98 m	91 m
Block partitioning	**1**:2:3:3	**1**:2:3:3	**1**:2:3:3	**1**:2:3:3	2:3:3:**1**	2:3:3:**1**
OOC (31,3,1)	95 m	105 m	105 m	98 m	105 m	98 m
PC p=5	89 m	98 m	98 m	92 m	98 m	92 m
MPC p=5,w=4	91 m	101 m	101 m	94 m	101 m	94 m
Block partitioning	**1**:4:4	**1**:4:4	**1**:4:4	**1**:4:4	4:4:**1**	4:4:**1**
OOC (31,3,1)	95 m	109 m	109 m	102 m	109 m	102 m
PC p=5	89 m	102 m	102 m	96 m	102 m	96 m
MPC p=5,w=4	91 m	105 m	105 m	98 m	105 m	98 m
Block partitioning	**1**:5:3	**1**:5:3	**1**:5:3	**1**:5:3	5:3:**1**	5:3:**1**
OOC (31,3,1)	95 m	109 m	109 m	102 m	109 m	102 m
PC p=5	89 m	102 m	102 m	96 m	102 m	96 m
MPC p=5,w=4	91 m	105 m	105 m	98 m	105 m	98 m
Block partitioning	**1**:8	**1**:8	**1**:8	**1**:8	8:**1**	8:**1**
OOC (31,3,1)	95 m	114 m	114 m	102 m	114 m	109 m
PC p=5	89 m	107 m	107 m	96 m	107 m	102 m
MPC p=5,w=4	91 m	110 m	110 m	98 m	110 m	105 m

**Table 15:** Accuracy as a combination of modulation and spreading code.

Spreading Codes	OOK	PPM	DPPM	MPPM	DPIM	DH-PIM${}_{2}$
Block partitioning	**1**:2:2:2:2	**1**:2:2:2:2	**1**:2:2:2:2	**1**:2:2:2:2	2:2:2:2:**1**	2:2:2:2:**1**
OOC (31,3,1)	29.19	30.16 mm	30.41 mm	29.19 mm	30.16 mm	29.51 mm
PC p=5	29.05 mm	29.72 mm	29.25 mm	29.05 mm	29.47 mm	29.05 mm
MPC p=5,w=4 mm	29.12 mm	29.99 mm	29.33 mm	29.21 mm	29.54 mm	29.64 mm
Block partitioning	**1**:2:3:3	**1**:2:3:3	**1**:2:3:3	**1**:2:3:3	2:3:3:**1**	2:3:3:**1**
OOC (31,3,1)	29.57 mm	30.60 mm	31.06 mm	29.61 mm	30.43 mm	29.93 mm
PC p=5	28.86 mm	29.59 mm	30.11 mm	29.28 mm	29.46 mm	29.31 mm
MPC p=5,w=4	29.45 mm	29.89 mm	30.06 mm	29.49 mm	30.03 mm	29.96 mm
Block partitioning	**1**:4:4	**1**:4:4	**1**:4:4	**1**:4:4	4:4:**1**	4:4:**1**
OOC (31,3,1)	29.43 mm	30.67 mm	32.36 mm	29.47 mm	30.62 mm	30.18 mm
PC p=5	29.28 mm	29.99 mm	30.29 mm	29.34 mm	30.73 mm	29.33 mm
MPC p=5,w=4	29.10 mm	30.36 mm	30.17 mm	29.26 mm	30.54 mm	29.48 mm
Block partitioning	**1**:5:3	**1**:5:3	**1**:5:3	**1**:5:3	5:3:**1**	5:3:**1**
OOC (31,3,1)	29.98 mm	31.14 mm	31.37 mm	30.14 mm	31.47 mm	30.64 mm
PC p=5	29.57 mm	30.44 mm	30.12 mm	29.23 mm	30.47 mm	30.27 mm
MPC p=5,w=4	29.66 mm	30.56 mm	30.91 mm	29.58 mm	30.91 mm	29.74 mm
Block partitioning	**1**:8	**1**:8	**1**:8	**1**:8	8:**1**	8:**1**
OOC (31,3,1)	29.92 mm	32.88 mm	32.31 mm	30.34 mm	32.82 mm	31.95 mm
PC p=5	29.11 mm	30.56 mm	31.13 mm	29.70 mm	30.93 mm	30.30 mm
MPC p=5,w=4	29.34 mm	31.54 mm	31.71 mm	29.55 mm	31.86 mm	31.05 mm

**Table 16:** Precision as a combination of modulation and spreading code.

Spreading Codes	OOK	PPM	DPPM	MPPM	DPIM	DH-PIM${}_{2}$
Block partitioning	**1**:2:2:2:2	**1**:2:2:2:2	**1**:2:2:2:2	**1**:2:2:2:2	2:2:2:2:**1**	2:2:2:2:**1**
OOC (31,3,1)	3.74 mm	4.86 mm	4.85 mm	3.74 mm	4.91 mm	3.60 mm
PC p=5	2.89 mm	3.93 mm	3.69 mm	2.89 mm	3.78 mm	2.87 mm
MPC p=5,w=4	3.12 mm	4.14 mm	4.03 mm	3.12 mm	4.41 mm	3.24 mm
Block partitioning	**1**:2:3:3	**1**:2:3:3	**1**:2:3:3	**1**:2:3:3	2:3:3:**1**	2:3:3:**1**
OOC (31,3,1)	3.69 mm	5.52 mm	5.52 mm	4.06 mm	5.58 mm	4.29 mm
PC p=5	2.83 mm	4.21 mm	4.30 mm	3.19 mm	4.27 mm	3.11 mm
MPC p=5,w=4	3.14 mm	4.85 mm	4.87 mm	3.42 mm	4.78 mm	3.69 mm
Block partitioning	**1**:4:4	**1**:4:4	**1**:4:4	**1**:4:4	4:4:**1**	4:4:**1**
OOC (31,3,1)	3.68 mm	6.24 mm	6.55 mm	5.05 mm	6.40 mm	5.03 mm
PC p=5	2.85 mm	5.03 mm	5.03 mm	3.76 mm	4.88 mm	3.80 mm
MPC p=5,w=4	3.25 mm	5.35 mm	5.49 mm	4.31 mm	5.62 mm	4.17 mm
Block partitioning	**1**:5:3	**1**:5:3	**1**:5:3	**1**:5:3	5:3:**1**	5:3:**1**
OOC (31,3,1)	3.68 mm	6.22 mm	6.39 mm	4.99 mm	6.13 mm	4.96 mm
PC p=5	2.81 mm	5.20 mm	4.97 mm	3.85 mm	5.25 mm	3.96 mm
MPC p=5,w=4	3.33 mm	5.52 mm	5.53 mm	4.24 mm	5.66 mm	4.32 mm
Block partitioning	**1**:8	**1**:8	**1**:8	**1**:8	8:**1**	8:**1**
OOC (31,3,1)	3.83 mm	7.82 mm	7.68 mm	4.92 mm	7.81 mm	6.51 mm
PC p=5	2.81 mm	6.05 mm	5.95 mm	3.86 mm	5.82 mm	5.04 mm
MPC p=5,w=4	3.23 mm	6.91 mm	6.77 mm	4.09 mm	6.97 mm	5.61 mm

**Table 17:** Error rate at maximum distance $R_{max}$ as a combination of modulation and spreading code.

Spreading Codes	OOK	PPM	DPPM	MPPM	DPIM	DH-PIM${}_{2}$
Block partitioning	**1**:2:2:2:2	**1**:2:2:2:2	**1**:2:2:2:2	**1**:2:2:2:2	2:2:2:2:**1**	2:2:2:2:**1**
OOC (31,3,1)	0.006 19	0.000 08	0.000 08	0.000 68	0.000 08	0.000 68
PC p=5	0.025 04	0.000 53	0.000 53	0.002 80	0.000 53	0.002 80
MPC p=5,w=4	0.014 15	0.000 26	0.000 26	0.001 58	0.000 26	0.001 58
Block partitioning	**1**:2:3:3	**1**:2:3:3	**1**:2:3:3	**1**:2:3:3	2:3:3:**1**	2:3:3:**1**
OOC (31,3,1)	0.006 19	0.000 03	0.000 03	0.000 30	0.000 03	0.000 30
PC p=5	0.025 04	0.000 26	0.000 26	0.001 45	0.000 26	0.001 45
MPC p=5,w=4	0.014 15	0.000 11	0.000 11	0.000 75	0.000 11	0.000 75
Block partitioning	**1**:4:4	**1**:4:4	**1**:4:4	**1**:4:4	4:4:**1**	4:4:**1**
OOC (31,3,1)	0.006 19	0.000 01	0.000 01	0.000 08	0.000 01	0.000 08
PC p=5	0.025 04	0.000 08	0.000 08	0.000 53	0.000 08	0.000 53
MPC p=5,w=4	0.014 15	0.000 03	0.000 03	0.000 26	0.000 03	0.000 26
Block partitioning	**1**:5:3	**1**:5:3	**1**:5:3	**1**:5:3	5:3:**1**	5:3:**1**
OOC (31,3,1)	0.006 19	0.000 01	0.000 01	0.000 08	0.000 01	0.000 08
PC p=5	0.025 04	0.000 08	0.000 08	0.000 53	0.000 08	0.000 53
MPC p=5,w=4	0.014 15	0.000 03	0.000 03	0.000 26	0.000 03	0.000 26
Block partitioning	**1**:8	**1**:8	**1**:8	**1**:8	8:**1**	8:**1**
OOC (31,3,1)	0.006 19	0.000 00	0.000 00	0.000 08	0.000 00	0.000 01
PC p=5	0.025 04	0.000 01	0.000 01	0.000 53	0.000 01	0.000 08
MPC p=5,w=4	0.014 15	0.000 00	0.000 00	0.000 26	0.000 00	0.000 03

## Abbreviations

ADC	Analog-to-digital converter
AMCW	Amplitude-modulated continuous wave
ASPRS	American Society for Photogrammetry and Remote Sensing
BER	Bit error rate
CID	Column identification number
CRC	Cyclic redundancy check
DPIM	Digital pulse interval modulation
DPPM	Differential pulse position modulation
DH-PIM	Dual-header pulse interval modulation
DPTM	Digital pulse time modulation
DSP	Digital signal processor
FMCW	Frequency-modulated continuous wave
GaAs	Gallium arsenide
GS	Guard slot
IEC	International electrotechnical commission
IM/DD	Intensity-modulated direct detection
LIDAR	Light detection and ranging
MEMS	Microelectromechanical systems
MPE	Maximum permissible rxposure
MPC	Modified prime code
MPPM	Multipulse pulse position modulation
MSB	Most significant bit
NLOS	Non-line of sight
NRZ	Non-return-to-zero
OCDMA	Optical code division multiple access
OOC	Optical orthogonal code
OOK	On–off keying
PC	Prime code
PER	Packet error rate
PIN	Positive–intrinsic–negative
PPM	Pulse position modulation
RID	Row identification number
RF	Radio frequency
RMSE	Root-mean-square-error
RZ	Return-to-zero
SER	Time slot error rate
SNR	Signal-to-noise ratio
TDMA	Time-division multiple access
TIA	TransImpedance amplifier
TNR	Threshold-to-noise ratio
ToF	Time-of-flight
WDMA	Wavelength-division multiple access
